# Treatment of T1b glottic SCC: laser vs. radiation- a Canadian multicenter study

**DOI:** 10.1186/1916-0216-42-22

**Published:** 2013-03-19

**Authors:** S Mark Taylor, Paul Kerr, Kevin Fung, Mankavil K Aneeshkumar, Derek Wilke, Yida Jiang, John Scott, Judith Phillips, Robert D Hart, Jonathan RB Trites, Matthew H Rigby

**Affiliations:** 1 Dalhousie University, Division of Otolaryngology – Head and Neck Surgery, Halifax, NS, Canada; 2 The University of Manitoba, Department of Otolaryngology – Head and Neck Surgery, Winnipeg, Canada; 3 Western University, Department of Otolaryngology – Head and Neck Surgery, London, ON, Canada; 4 Dalhousie University, Department of Radiation Oncology, Halifax, NS, Canada; 5 Otolaryngology - Head & Neck Surgery Facial Plastic & Reconstructive Surgery, Suite 3052, Dickson Bldg., 5820 University Avenue, Halifax, NS B3H 1V9, Canada

**Keywords:** Anterior commissure, SCC, Glottis, Laser, Radiation

## Abstract

**Objective:**

To assess the oncological and functional outcomes of T1b squamous cell carcinoma (SCC) of the glottic larynx treated with laser in comparison with radiation.

**Design:**

A Canadian multicenter cohort study.

**Setting:**

Three tertiary referral centers for head and neck cancer- Dalhousie University in Halifax, Nova Scotia, Western University in London, Ontario and the University of Manitoba, Winnipeg.

**Methods:**

Patients with T1b glottic SCC who underwent transoral laser resection or radiation as the primary modality of treatment.

**Outcome measures:**

Oncological outcomes were evaluated using local control, laryngeal preservation, disease free survival and disease specific survival. Voice outcomes were assessed using the Voice Handicap Index-10 (VHI-10).

**Results:**

63 patients met study criteria. 21 were treated with laser and 42 with radiation. Oncologic outcomes at 2 years for laser and radiation demonstrated local control of 95% and 85.9%; laryngeal preservation of 100% and 85.9%; disease free survival of 88.7% and 85.9% and overall survival of 94.1% and 94.8% respectively. VHI-10 data was available for 23/63 patients. During the last follow up visit VHI-10 ranged from 0 to 11 (median 6) in the laser group and 0 to 34 (median 7) in the radiation group.

**Conclusion:**

T1b SCC of the glottis can be effectively treated with transoral laser microsurgery with oncological outcomes that are at least equivalent to radiation. For patients with VHI scores, voice quality was similar between the two groups. To our knowledge this is the first study directly comparing the oncologic and voice outcomes with laser and radiation for the treatment of glottic cancer involving the anterior commissure.

## Introduction

The last decade has seen a significant change in the management of laryngeal cancer. In the past, early laryngeal cancers have traditionally been treated with radiation or open partial laryngeal surgery. Transoral laser microsurgery for early stage glottic cancer was originally described by Strong and Jako in 1972 [1]. This was further popularized by Steiner [2]. With the knowledge gained from this experience laser has been recently used for the management of locally advanced laryngeal cancer [3]. The popularity of laser surgery has also resulted in a significant decline in open partial surgery.

Anterior commissure involvement is seen in about 20% of all glottic tumours and is generally associated with worse outcomes [4]. Some consider Broyle’s ligament as a barrier for tumour spread while other consider this as an easy access for tumour to spread to the thyroid cartilage [5-7]. Many believe that voice outcome after resection of the anterior commissure with laser is worse than treating with radiation. There is no conclusive evidence on the superiority of voice outcomes with either treatment and the current literature reports mixed results. Uncertainty regarding the voice outcomes and the difficulty in gaining access to the anterior commissure are factors that prevent some surgeons from attempting laser resection of these lesions. In our study, we assessed the oncological and voice outcomes after laser resection and radiation of tumours involving this very complex area.

## Materials and methods

All patients with primary T1bN0M0 squamous cell carcinoma of the glottis presenting to three tertiary referral centres in Canada from 2002 to 2010 were included in the study. Patients were staged using endoscopy (flexible or rigid) and, in some instances scanning (MRI/CT). Recurrent tumours and tumours that were not staged at T1b were excluded from the present analysis. All patients were presented at the multidisciplinary tumour board at their respective institutions. In all the three centers the tumour board recommendation and the options of treatment with surgery and radiation were discussed with the patient by the surgical oncologist and radiation oncologist. After meeting with both specialists, patients chose their treatment modality based on personal preferences and tumour board recommendation.

Oncological and functional outcomes data were prospectively collected by each center independently. Approval for data collection was obtained in advance by each institutional research ethics board. Oncological outcome measures used were local control, laryngeal preservation, disease free survival and overall survival. Voice outcomes were measured using the voice handicap index-10 (VHI-10). VHI-10 data was not originally collected at every center, and collection was standardized midway through the study. Table 1 shows the VHI-10 scoring sheet.

**Table 1:** VHI-10 score


F 1. My voice makes it difficult for people to hear me.	*0*	*1*	*2*	*3*	*4*
P 2. I run out of air when I talk.	*0*	*1*	*2*	*3*	*4*
F 3. People have difficulty understanding me in a noisy room.	*0*	*1*	*2*	*3*	*4*
P 4. The sound of my voice varies throughout the day.	*0*	*1*	*2*	*3*	*4*
F 5. My family has difficulty hearing me when I call them throughout the house.	*0*	*1*	*2*	*3*	*4*
F 6. I use the phone less often than I would like to.	*0*	*1*	*2*	*3*	*4*
E 7. I’m tense when talking to others because of my voice.	*0*	*1*	*2*	*3*	*4*
F 8. I tend to avoid groups of people because of my voice.	*0*	*1*	*2*	*3*	*4*
E 9. People seem irritated with my voice.	*0*	*1*	*2*	*3*	*4*
P 10. People ask, “What’s wrong with your voice?”	*0*	*1*	*2*	*3*	*4*

0 = never, 1 = almost never, 2 = sometimes, 3 = almost always, 4 = always.

A descriptive analysis of demographics, morbidities, outcomes and VHI-10 data was performed. SPSS version 17 was used for the analysis. Student’s *t*-test was used to analyse continuous, normally distributed variables and Fisher’s exact test was used to analyse nominal variables. All statistical testing was performed using an intention to treat analysis unless otherwise stated in the results. Kaplan Meier 24-month survival analyses were performed for: local control, disease free survival, laryngeal preservation, and overall survival. An event for local control was defined as a local recurrence obtained after TLM. Disease free survival was defined as either local or regional recurrence or the presence of a second glottic primary. Cancers were defined as a second primary if they occurred greater than 5 years after the last received treatment modality, or if they occurred on the contralateral side to a previously treated unilateral tumour that not cross midline and did not involve the anterior commissure.

## Results

A total of 63 patients were included in the study. Of these, 21 patients underwent laser resection and 42 patients received radiation. All patients in the radiation arm were treated with a planned curative dose of radiation. Follow up ranged from 5 to 102 months (median 34 in both laser and RT groups). Of the 63 patients included in the study, there were 57 males and 6 females. See Table 2 for demographic information.

**Table 2:** Demographic information

**Variable**	**TLM**	**Radiation**	**p**
**Age (mean)**	64.3	68.6	0.15
**Gender (male/female)**	18/3	39/3	0.39
**Follow up (months - mean)**	35.6	35.4	0.96

Three patients in the laser group developed complications. One patient developed a vocal cord granuloma that was managed conservatively and after 6 years his VHI score was 5. This patient is currently singing in a traditional music group and is pleased with their postoperative voice. Another patient developed an anterior glottic web and underwent division of the web and insertion of a laryngeal keel. This patient’s VHI score was 21 prior to the reconstructive surgery but was reduced to 10 at the time of his last visit.

No patients in the laser group required a tracheostomy. One patient developed respiratory distress after surgery and had to be re-intubated. He was successfully extubated after 24 hours in ICU. There were two complications in the radiotherapy group. One patient developed significant chondronecrosis and required a functional total laryngectomy. A second patient required a tracheostomy due to airway compromise.

### Oncologic outcomes

**Figure 1:**
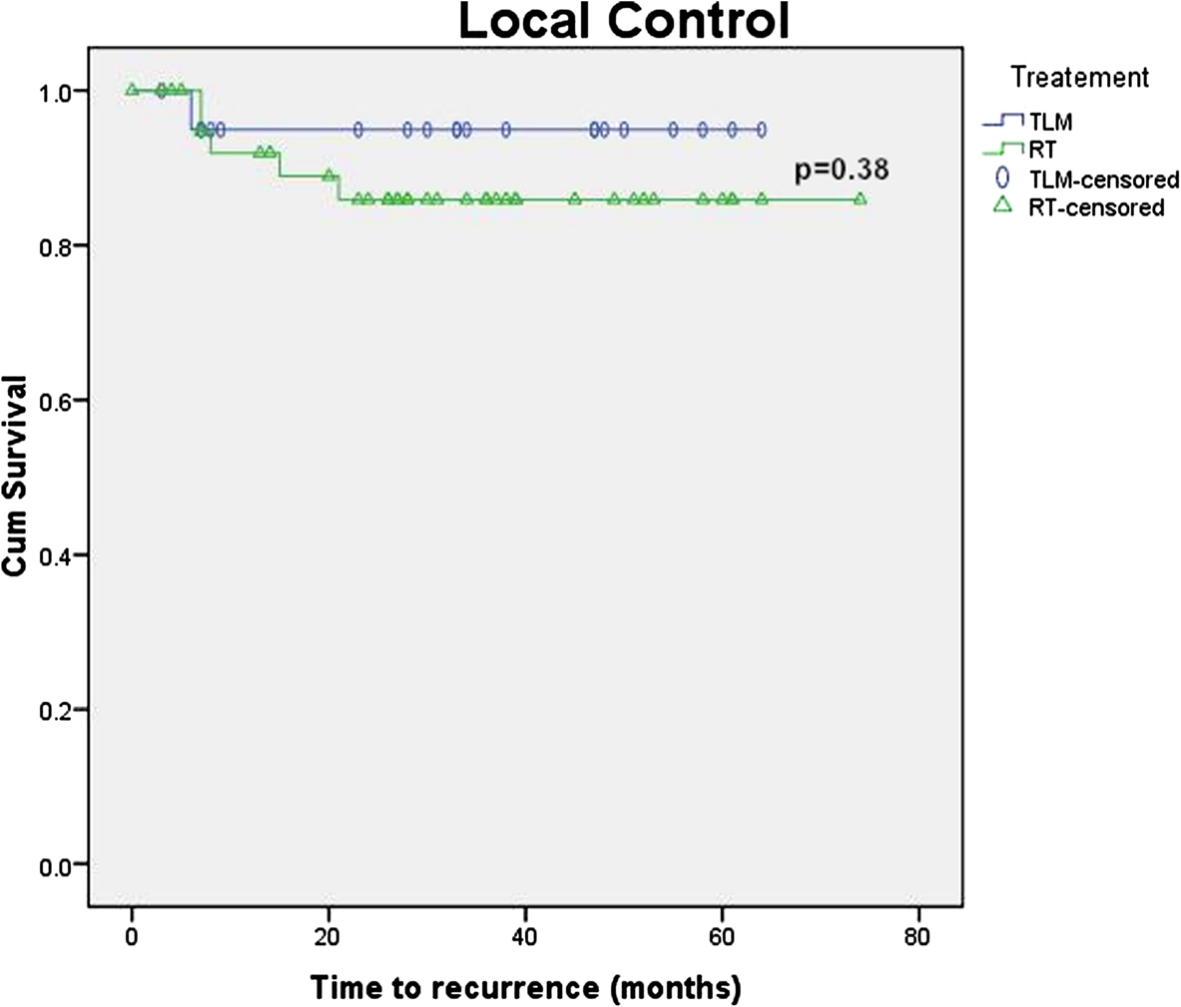
Local control.

**Figure 2:**
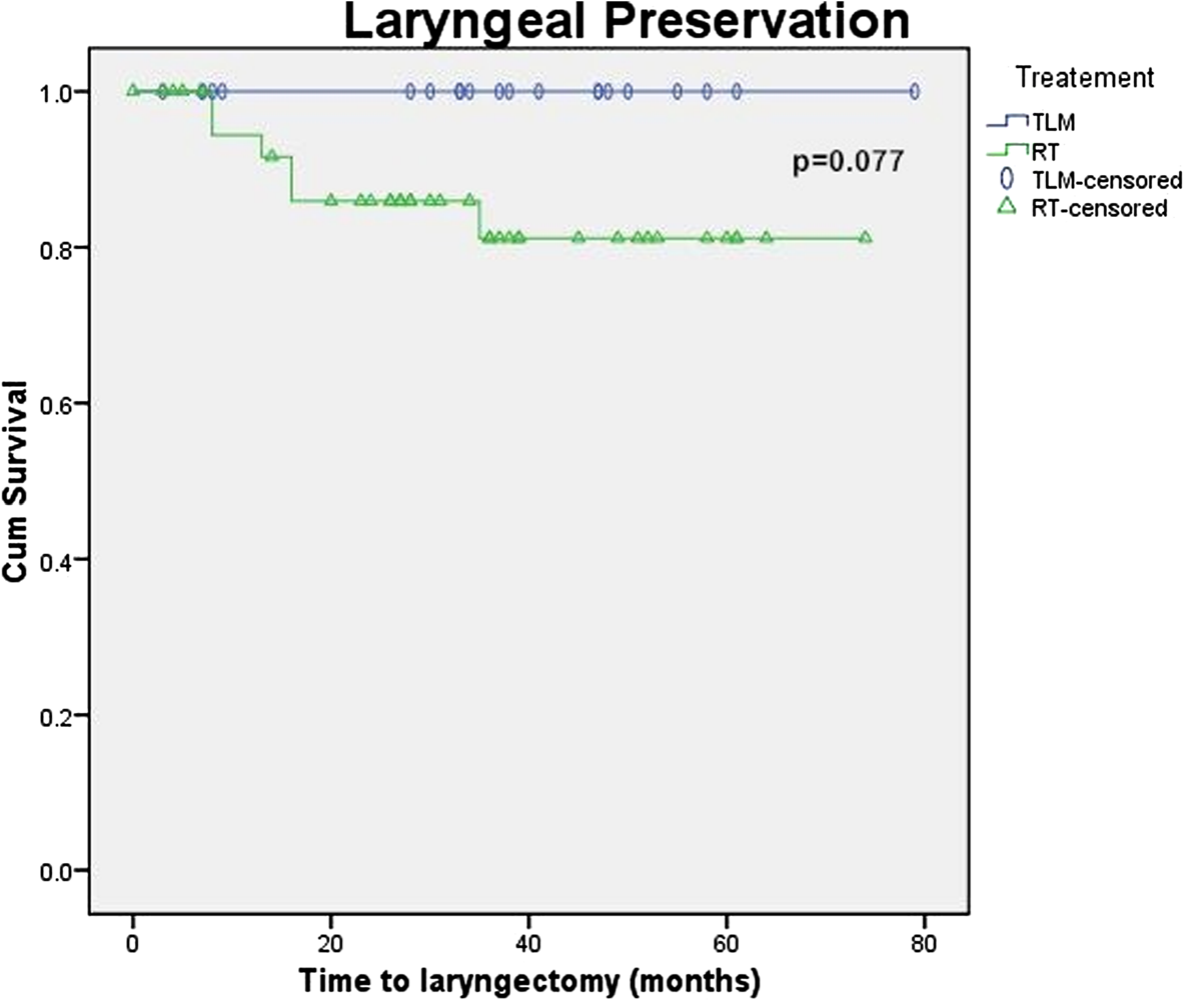
Laryngeal preservation.

**Figure 3:**
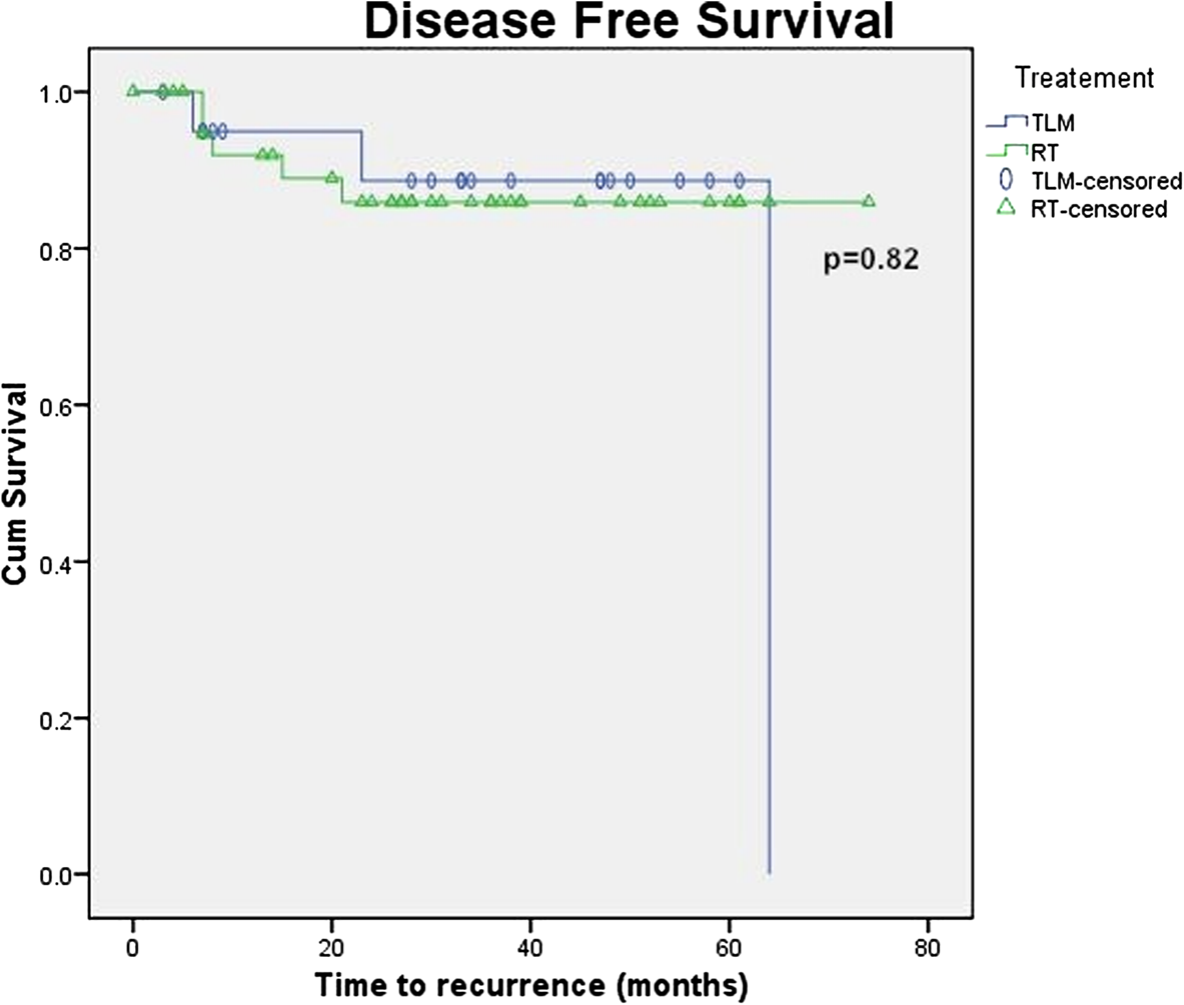
Disease free survival.

**Figure 4:**
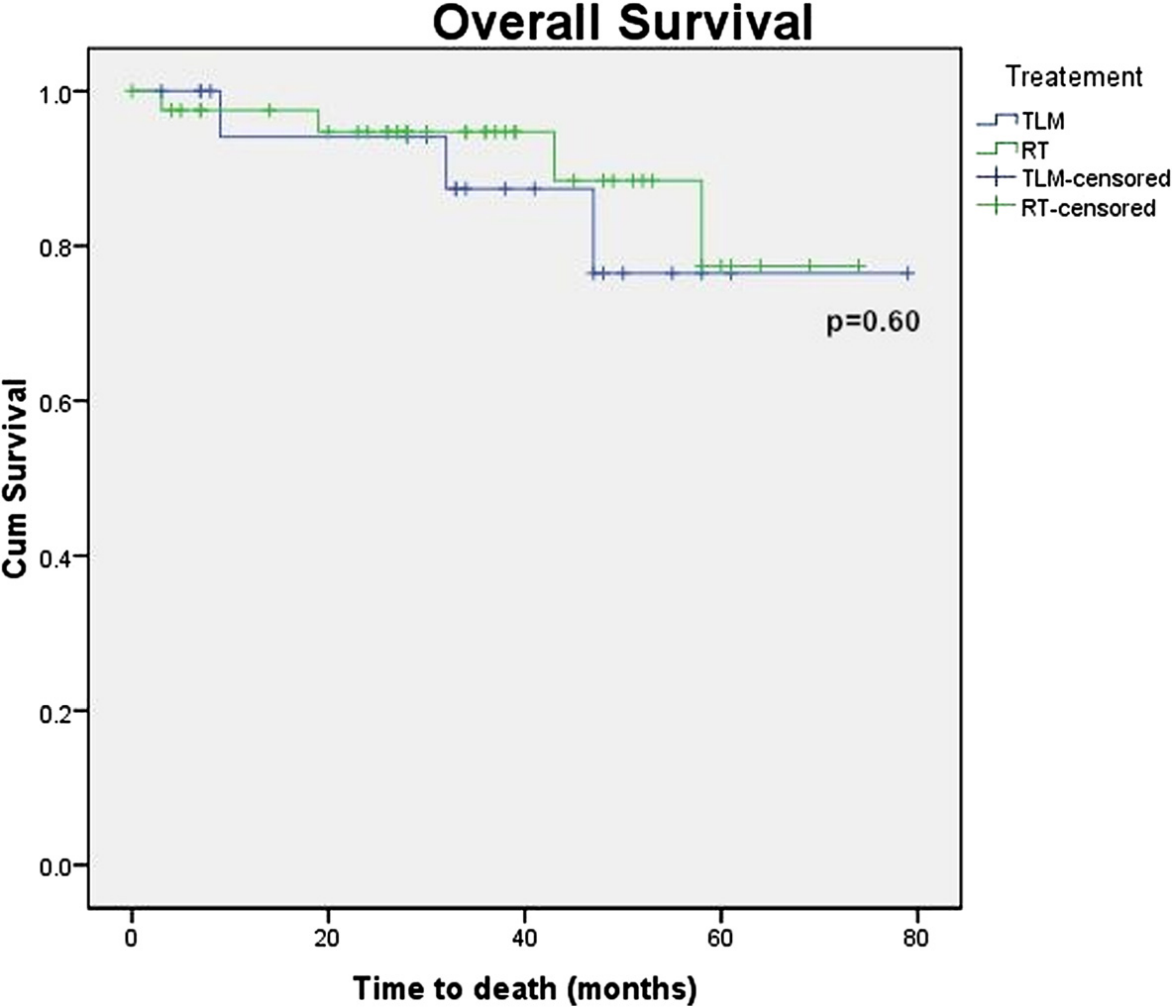
Overall survival.

Figures 1, 2, 3 and 4 Kaplan Meier plots for the local control, laryngeal preservation, disease free survival and overall survival. Two patients recurred in the laser arm at 6 and 23 months respectively. The patient who recurred after 6 months had aggressive disease when the recurrence was diagnosed. The recurrence was in the contralateral vocal cord (cord with minimal disease at the time of the primary treatment) with metastatic neck nodes in the ipsilateral side. This was salvaged with neck dissection, further laser treatment and post-operative chemoradiation. The patient who recurred at 23 months developed regional neck node metastasis in the pretracheal and supraclavicular nodes and distant pulmonary metastasis but no local recurrence. This patient underwent treatment with palliative intent and died 37 months after the primary treatment. One patient died due to causes unrelated to the tumour. The two-year survival rates are as follows: local control rate of 95%, laryngeal preservation rate of 100% disease free survival of 88.7% and overall survival of 94.1%.

Five patients in the radiation group developed local recurrence at 6, 7, 8, 15 and 21 months after the primary treatment. Four patients underwent salvage total laryngectomy of which one patient died of peristomal recurrence. One patient was salvaged with open partial laryngectomy. A single patient died due to tumour while 3 patients died due to factors not related to their laryngeal carcinoma. The two-year survival rates are as follows: local control rate of 85.9%, laryngeal preservation rate of 85.9%, disease free survival of 85.9%, and overall survival of 94.8%.

### Voice outcomes

VHI-10 data was available for 13 patients in radiation group and 10 patients in laser group. During the last work up VHI-10 ranged from 0 to 11 (median 6) in the laser group and 0 to 34 (median 7) in the radiation group. In the radiation group one patient had a tracheostomy (VHI-10 of 34), which resulted in a wider range of VHI score.

## Discussion

Staging of the involvement of anterior commissure is controversial. Steiner has further classified T1a, T1b and T2a, as with or without anterior commissure involvement [8]. There is no international consensus on the management of tumours involving the anterior commissure. Certain countries have issued guidelines for the management of tumours involving the anterior commissure. Dutch national guidelines recommend performing laser surgery only on T1a lesions when a sufficient margin can be obtained within the affected fold. They further recommend this using either a subligamental or subepithelial resection that does not extend into the anterior commissure. Lesions that require resection of the anterior commissure have in the past largely been regarded as unsuitable for laser surgery because of poor voice quality [9]. According to the consensus statement on management in the United Kingdom, patients should be given the choice of TLM for tumours involving the anterior commissure, but advised of the greater chance of adverse voice outcome when the anterior commissure is treated surgically [10]. Steiner and colleagues reported that laser surgery is effective despite anterior commissure involvement [11].

Disadvantages of laser surgery include: the need for general anaesthesia, difficult access to anterior commissure, and possibly poor voice outcome due to resection of anterior commissure. By comparison the disadvantages of radiotherapy include: longer treatment schedule, increased expense, risk of chondronecrosis, development of radiation induced tumours, delay in diagnosing recurrence due to oedema, and total laryngectomy is often required as a salvage operation [12].

### Oncological outcomes

According to some studies, anterior commissure involvement adversely affects local control, laryngeal preservation and survival [13-15]. Other studies have not found this effect to be significant [16,17]. Mlynarek compared patients with early glottic cancer after radiotherapy and microsurgery and found that in both groups 50% of patients with involvement of anterior commissure had recurrences [18].

Opinion is divided regarding the effectiveness of radiotherapy for local control of tumours involving the anterior commissure. Five-year control rates for tumours with involvement of the anterior commissure varied from 56% to 80% and without anterior commissure involvement from 82% to 90% [13,14,19,20]. In contrast a study by Mendenhall showed no difference in local control rate [21]. Sjogren studied 36 patients with anterior commissure involvement who had radiotherapy as their primary treatment. Five patients developed local recurrence and 1 patient developed distant metastasis. Three patients underwent total laryngectomy as salvage. Their local control and laryngeal preservation rates were 88% and 91% respectively [9].

Steiner and colleagues studied 89 T1 glottic cancer patients with anterior commissure involvement and reported 21 local recurrences. Their 5 year local control, laryngeal preservation rate, ultimate local control and overall survival were 71%, 95%, 98% and 88% respectively [8]. In a larger series by Motta (169 patients with anterior commissure involvement) actuarial survival, adjusted actuarial survival and ultimate local control were 84%, 96% and 83% respectively [22]. Gallo studied 22 patients with anterior commissure involvement and local control and overall survival were 91% and 95% respectively [23]. In the paper by Bocciolini 5 out of 10 patients developed local recurrence and their laryngeal preservation rate was 80%.

In our study there was an apparent difference in the local control and laryngeal preservation rates between the laser and radiation arms as seen from the Kaplan Meier plots. These observed differences would be clinically significant, but are not statistically significant in the present study (p = 0.38 for local control and 0.077 for laryngeal preservation using the Log Rank test). In the present study, the lack of significance likely represents our small sample size rather than no true effect. Disease free survival and overall survival shows no significant differences between modalities (p = 0.82 and 0.6 respectively using the Log Rank test).

### Voice outcomes

Cohen in 2006 performed a meta-analysis from 1966 to 2005 comparing voice outcomes with radiation compared with laser for the treatment of early glottic cancer. This series included 6 studies with 208 patient (6 T1b and 202 T1a) treated with laser and 91 patients (6T1b and 85 T1a) treated with radiation. This showed comparable levels of voice handicap with both interventions. This study reported that the resections involving the superficial vocalis muscle (mean VHI, 6.23) had improved VHI scores compared to those involving the contralateral vocal fold (mean VHI, 15.7) and concluded that further study is needed to clarify voice outcomes in lesions involving anterior commissure [24]. Our study shows no obvious difference in the post-operative VHI-10 score between the two arms.

To our knowledge there are no studies in the literature directly comparing the oncologic and voice outcomes for the treatment of glottic cancer with anterior commissure involvement after treatment with laser and radiation. Access to the anterior commissure is one of the determining factors when assessing the feasibility for laser resection. We generally use Kleinsasser laryngoscope to expose the larynx. In our experience we were able to successfully complete the procedure in all the patients listed for laser resection in the T1b cohort.

### Limitations of the study

Due to lack of randomization study design the possibility of selection bias is unavoidable. Given that all tumours were a narrowly defined T-stage and demographic information was similar between groups, we do not believe that this bias is so significant that it negates our ability to make meaningful comparisons between groups.

A further weakness of the study was the lack universal VHI-10 data. Finally, lack of intra-operative staging in the radiation arm could have potentially under staged some cancers. Interestingly none of the patients in the laser arm were up staged intra-operatively.

## Conclusion

In our experience stage T1b squamous cell carcinoma of the glottis can be effectively treated with transoral laser microsurgery with oncological outcomes that appear to be superior when compared to primary radiation. Post-therapy voice quality, which has been a major consideration in the selection of treatment modality, was similar in both groups. To our knowledge this is the first study directly comparing the oncologic and voice outcomes with laser and radiation for the treatment of glottic cancer involving the anterior commissure.

## Competing interests

The authors declare that they have no competing interests.

## Authors’ contributions

SMT, PK, KF, MHR, MKA, DW, RDH and JRBT were involved in study design. MHR, MKA, YJ, JS and JP were involved in data collection. MHR performed the statistical analysis and interpretation. SMT, MHR and MKA were involved in drafting and revising the manuscript. All authors read and approved the final manuscript.

This paper was awarded the top paper in Head and Neck Surgery at the Canadian Society of Otolaryngology Head and Neck Surgery Conference on the 21st of May 2012 at Toronto.
